# The Elements of Ophthalmoscopic Diagnosis

**Published:** 1891-06

**Authors:** 


*The Elements of Ophthalmoscopic Diagnosis.* By GEORGE A. BERRY, M.B., F.R.C.S. Ed. Edinburgh: YOUNG J. PENTLAND. 1891.

If it be an advantage to the student for a time to concentrate his attention upon what he can learn through the ophthalmoscope, the book before us will certainly help him to systematise his work, and will save him the trouble of searching through the text-books for the information he requires. One sufficiently skilled in the use of the ophthalmoscope, with the material afforded by a large eye clinic, might feel sure that Berry's book would help him rapidly and thoroughly to traverse the subject with which it deals.

The appearances of the normal fundus, and the exceptional physiological peculiarities or congenital anomalies met with, are first described. Then the various affections of the retina, choroid, and optic nerve are depicted in clear and accurate language.

Opacities in the media—vitreous, lens, and cornea—are also described.

The value of direct examination of the cornea, with help of a strong lens (20 D), is not mentioned: by this means slight keratitis punctata, or lens opacities, are most readily detected. There are but few omissions, and no errors; and the book fairly covers the elements of ophthalmoscopic diagnosis, and is worthy of the high reputation of its author.

